# Study on Chaotic Fault Tolerant Synchronization Control Based on Adaptive Observer

**DOI:** 10.1155/2014/405396

**Published:** 2014-05-18

**Authors:** Dongming Chen, Xinyu Huang, Tao Ren

**Affiliations:** Software College, Northeastern University, Shenyang 110819, China

## Abstract

Aiming at the abrupt faults of the chaotic system, an adaptive observer is proposed to trace the states of the master system. The sufficient conditions for synchronization of such chaotic systems are also derived. Then the feasibility and effectiveness of the proposed method are illustrated via numerical simulations of chaotic Chen system. Finally, the proposed synchronization schemes are applied to secure communication system successfully. The experimental results demonstrate that the employed observer can manage real-time fault diagnosis and parameter identification as well as states tracing of the master system, and so the synchronization of master system and slave system is achieved.

## 1. Introduction

Chaotic systems are complex nonlinear systems whose behaviors are highly sensitive to initial conditions, and they are long-term unpredictable in general, resulting long-term unpredictable in general. The characteristics of chaos have been attracting researchers' attention since 1990s. The realization of OGY (Ott-Grebogi-Yorke) chaos-control method [1] and PC synchronization method (Pecora and Carroll's method) [2] had greatly promoted the application of chaos theory. As the key technology for secure communications, chaotic synchronization research has made some progress [3–14].

For many dynamic systems, chaotic systems, for example, system parameters and models, may change with the environments. Because of the uncertainty of these parameters or model, it is usually very difficult to synchronize the slave system and the master system. However, observer-based synchronization control methods need not know all statuses of the original chaotic system, so it is more suitable for practical applications [15–20]. Paper [15] gave the conditions that observers exist, proposed a specific form of observer, and applied the designed synchronization scheme to chaotic secure communication systems. Paper [16] researched the scheme of exponential synchronization for a class of time-delay Chen chaotic systems with unknown parameters, designed a synchronization controller and adaptive laws for parameters, and gave sufficient conditions of exponential synchronization. Paper [17] transforms the chaotic synchronization into the design of a special observer and realized the tracking of chaotic system statuses. Paper [18] designed the high precision secure communication scheme of hyperchaotic system based on the state observer method. Paper [19] achieved chaotic synchronization based on extended state observer and sliding model theory. Paper [20] designed a nonlinear observer based on Chua's circuit to achieve chaotic synchronization.

Works described above did not take system failures into account, and they all focused on the design of synchronization observers under normal operating conditions, while, in practical chaotic secure communication systems, sensors, actuators, and inner components may inevitably fail, which will lead to sharp performance decline of the chaotic secure circuits, and may even cause paralysis of the whole communication system and then lead to enormous economic losses. For this reason, the reliability of chaotic system is crucial in secure communication systems. Research on fault tolerant synchronization has become a hot topic in chaotic synchronization research area, and it is attracting more and more attentions.

Our work considers the abrupt fault in chaotic system, employs an observer-based active fault tolerant approach, diagnoses fault online using adaptive observer, and achieves synchronization of chaotic system effectively. We verified the effectiveness of our method through numerical simulations; experimental results showed that synchronization can be achieved no matter the system failed or not. Finally, we also applied our method to chaotic secure communications.

The rest of this paper is organized as follows.  Section 2 brings forward and formulates the problem.  Section 3 proposes a fault tolerant synchronization control scheme based on adaptive observer.  Section 4 demonstrates numerical simulations and applications for secure communications. Finally, Section 5 concludes this paper.

## 2. Problem Description

Consider the following chaotic system as a master system:

(1) $$\begin{array}{c} \dot{x}\left(t\right)=A\left(x\right)+Bg\left(x\left(t\right)\right)+Ff\left(t\right),\\ y\left(t\right)=Cx\left(t\right), \end{array}$$

where *x*(*t*) ∈ *R* ^*n*^ is the state variable of the system, *y*(*t*) ∈ *R* ^*n*^ is the output variable, *g*(·) is a nonlinear function, *A*, *B*, *C*, and *F* are coefficient matrices with appropriate dimensions, $f(t)=\{\begin{array}{cc} 0 & t<T\\ a & t\geq T \end{array}$ is the fault function, and *T* is the time of failure.

Observer-based chaotic synchronization method builds observer for the master system, and the constructed observer is employed as slave system to trace the state of the master system, and then the synchronization of master system and slave system is achieved. The adaptive observer is designed as

(2) $$\begin{array}{c} \dot{\hat{x}}\left(t\right)=A\dot{x}+Bg\left(\hat{x}\left(t\right)\right)+Ff\left(t\right)+0.5B\hat{\theta }\left(t\right)\left(y\left(t\right)-C\hat{x}\left(t\right)\right),\\ \hat{y}\left(t\right)=C\hat{x}\left(t\right), \end{array}$$

where *x*(*t*) and $\hat{y}(t)$ are estimate values of *x*(*t*) and *y*(*t*), respectively. $\hat{\theta }(t)$ is the gain of the observer with adaptive law and it satisfies the following adaptive law:

(3) $$\begin{array}{c} \dot{\hat{\theta }}\left(t\right)=k{||\left(y\left(t\right)-C\hat{x}\left(t\right)\right)||}^{2}. \end{array}$$

$\hat{f}(t)$ is the estimation of the system fault function *f*(*t*), and it satisfies the following adaptive law:

(4) $$\begin{array}{c} \dot{\hat{f}}\left(t\right)=\Gamma R\left(y\left(t\right)-C\hat{x}\left(t\right)\right), \end{array}$$

where Γ is a positive definite diagonal matrix, which is formulated as *R* = *F* ^*T*^(*B* ^−1^)^*T*^.

Assuming that the error of the fault function and the synchronization error of the chaotic system are, respectively, defined as

(5) $$\begin{array}{c} e_{f}\left(t\right)=f\left(t\right)-\hat{f}\left(t\right), \end{array}$$

(6) $$\begin{array}{c} e\left(t\right)=x\left(t\right)-\hat{x}\left(t\right), \end{array}$$

we can get the following error system from the derivative of (6):

(7) e˙(t)=Ae(t)+B(g(x^(t))−g(x^(t)))+Fef(t)−0.5Bθ^(t)(y(t)−Cx^(t))=(A−LC)e(t)+LCe(t)+B(g(x(t))−g(x^(t)))+Fef(t)−0.5Bθ^(t)(y(t)−Cx^(t)).

In order to facilitate design, we provide the following hypothesis and lemma.

**Hypothesis 1**

To chaotic system formulated in (1), there exist matrices *L*, *P* = *P* ^*T*^ > 0 and *Q* > 0, which satisfy the following equation:

(8) $$\begin{array}{c} P\left(A-LC\right)+{\left(A-LC\right)}^{T}P=-Q,\\ B^{T}P=C. \end{array}$$

**Hypothesis 2**

To chaotic system formulated in (1), nonlinear function *g*(·) satisfies the Lipschitz conditions; that is, for *x*(*t*) ∈ *R* ^*n*^, *y*(*t*) ∈ *R* ^*n*^, there exists *δ* > 0 satisfying the following inequality:

(9) $$\begin{array}{c} ||g\left(x\left(t\right)\right)-g\left(y\left(t\right)\right)||\leq \delta ||x\left(t\right)-y\left(t\right)||. \end{array}$$

**Lemma 1**

To appropriately dimensioned matrices *X*, *Y* and a positive definite matrix *R*, the following inequality is satisfied:

(10) $$\begin{array}{c} XY+Y^{T}X^{T}\leq XRX^{T}+Y^{T}R^{-1}Y. \end{array}$$

## 3. Fault Tolerant Synchronization Control Scheme

**Theorem 2**

To adaptive observer (2), there exist symmetric matrices *P* > 0, *Q* > 0, *L* and constants *α*, *β*; the following matrix inequality is satisfied:

(11) $$\begin{array}{c} Q-\beta PLL^{T}P-\alpha I>0, \end{array}$$

whereas error system (7) is asymptotic stability; that is, master system (1) and slave system (2) can be synchronized.

**Proof**

Select the following Lyapunov function:

(12) $$\begin{array}{c} V\left(t\right)=e^{T}\left(t\right)Pe\left(t\right)+\frac{1}{2k}{\overset{-}{\theta }}^{2}+e_{f}^{T}\left(t\right)\Gamma^{-1}e_{f}\left(t\right), \end{array}$$

where *P* is a positive definite symmetric matrix and it satisfies Hypothesis 1, $\overset{-}{\theta }=\theta -\hat{\theta }$ and *θ* = (1/*β*) + (2/*α*)*δ* ^2^ > 0, simultaneously.

From the derivative of *V*(*t*), we can get

(13) V˙(t)=2eT(t)Pe˙(t)+1kθ− θ−˙+2efT(t)Γ−1e˙f(t)=eT(t)(P(A−LC)+(A−LC)TP)e(t)+2eT(t)PLCe(t)+2eT(t)PB(g(x(t))−g(x^(t)))+2eT(t)PFef(t)−eT(t)PBθ^(t)(y(t)−Cx^(t))−1kθ− θ−˙−2efT(t)Γ−1f^˙(t).

According to Hypothesis 2 and Lemma 1, we have

(14) 2eT(t)PB(g(x(t))−g(x^(t))) ≤||2eT(t)PB(g(x(t))−g(x^(t)))|| ≤||2eT(t)PB||·||g(x(t))−g(x^(t))|| ≤2δ||eT(t)PB||·||e(t)|| ≤δ2α||eT(t)PB||2+α||e(t)||2 =δ2αeT(t)PBBTPe(t)+α||e(t)||2.

The following inequality can be derived from Hypothesis 1 and Lemma 1:

(15) 2eT(t)PLCe(t)=2eT(t)PLBTPe(t)≤βeT(t)PLLTPe(t)+1βPBBTPe(t).

From Hypothesis 1, we get

(16) 2eT(t)PFef(t)−2efT(t)Γ−1f^˙(t) =2eT(t)PFef(t)−2eT(t)Γ−1ΓR(y(t)−Cx^(t)) =2eT(t)PFef(t)−2efT(t)RCe(t) =2eT(t)PFef(t)−2efT(t)FT(B−1)TBTPe(t)=0.

Substitute (14), (15), and (16) into (13), the following inequality can be deduced:

(17) V˙(t)≤−eT(t)Qe(t)+βeT(t)PLLTPe(t)+α||e(e)||2+(1β+2αδ2−θ^(t))||BTPe(t)||2−1kθ− θ^˙≤−eT(t)(Q−βPLLTP−αI)e(t)+(θ−θ^(t))||BTPe(t)||2−1kθ− θ^˙.

Substituting adaptive law (3) and (4) into (17), the following inequality is reached:

(18) $$\begin{array}{c} \dot{V}\left(t\right)\leq -e^{T}\left(t\right)\left(Q-\beta PLL^{T}P-\alpha I\right)e\left(t\right). \end{array}$$

From Theorem 2, we conclude that inequality *Q* − *βPLL* ^*T*^ *P* − *αI* > 0 can be satisfied if we select *α* and *β* which are small enough. That is, error system is asymptotic stability when $\dot{V}(t)<0$; synchronization of master system and slave system is achieved. So only when we adjust the coefficients of *α*, *β* in (18) can we get *Q* − *βPLL* ^*T*^ *P* − *αI* > 0 to make the error system asymptotic stability.

## 4. Simulations and Applications for Secure Communications

In order to validate the correctness of Theorem 2, we select the following coefficient matrices and fault functions:

(19) $$\begin{array}{c} A=\left[\begin{array}{ccc} -35 & 35 & 0\\ -7 & 28 & 0\\ 0 & 0 & -3 \end{array}\right],\;\;B=\left[\begin{array}{ccc} 1 & 0 & 0\\ 0 & 1 & 0\\ 0 & 0 & 1 \end{array}\right],\\ C=\left[\begin{array}{ccc} 10 & 0 & 0\\ 0 & 10 & 0\\ 0 & 0 & 10 \end{array}\right],\;\;F=\left[\begin{array}{c} 0\\ 0\\ 2 \end{array}\right],\\ f\left(t\right)=\{\begin{array}{cc} 0, & t<5,\\ 5, & t\geq 5. \end{array} \end{array}$$

Let *k* = 1, Γ = 1, *α* = 0.01, and *β* = 0.01. Matrices *P*, *L*, *Q*, and *R* are presented as

(20) $$\begin{array}{c} P=\left[\begin{array}{ccc} 10 & 0 & 0\\ 0 & 10 & 0\\ 0 & 0 & 10 \end{array}\right],\\ Q=\left[\begin{array}{ccc} 130.4627 & 0 & 0\\ 0 & 130.4627 & 0\\ 0 & 0 & 130.4627 \end{array}\right],\\ L=\left[\begin{array}{ccc} -2.8142 & 1.300 & 0\\ 1.4575 & 3.3901 & 0\\ 0 & 0 & 0.3372 \end{array}\right],\;\;R=\left[0\;0\;2\right]. \end{array}$$

Let the initial state of the master system-equation (1) and the observer-equation (2) be formulated as *x*(*t*) = [0.5 0.2 0.5] and $\hat{x}\left(t\right)={\left[0.01\;-0.5\;0.1\right]}^{T}$, respectively.

**Figure 1:**
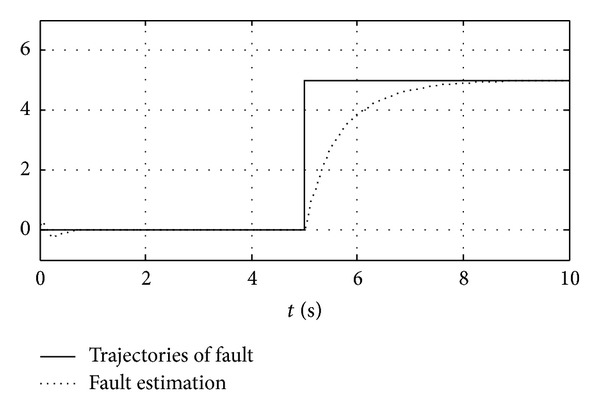
The trajectories of a fault (solid line) and the fault estimation (dotted line).

The trajectories of a fault and the fault estimation of the system are shown in Figure 1, where an abrupt fault occurs when *t* = 5 s. The adaptive observer estimates the faults within a short time after the fault occurs.

**Figure 2:**
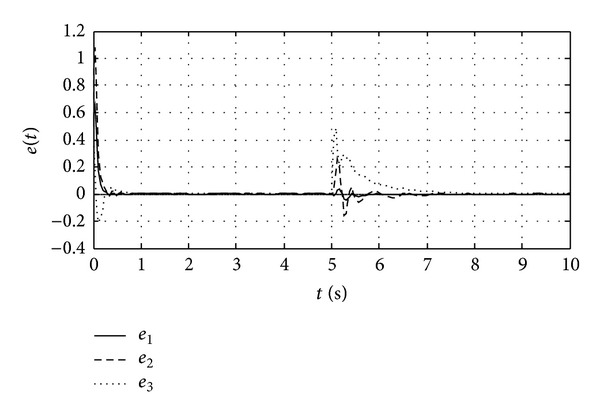
Three error trajectories between synchronized chaotic systems.

Figure 2 discloses the state trajectories of the error system. Error system turns stable after about 1 second under the circumstances of no faults occurring, so the master system and the slave system are comparatively rapidly synchronized. Abrupt fault of the master system occurs when *t* = 5 s, and the error system restabilizes within 3 seconds.

**Figure 3:**
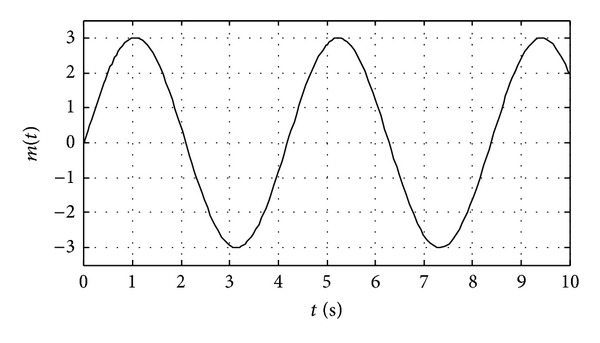
The original signal from transmitter.

**Figure 4:**
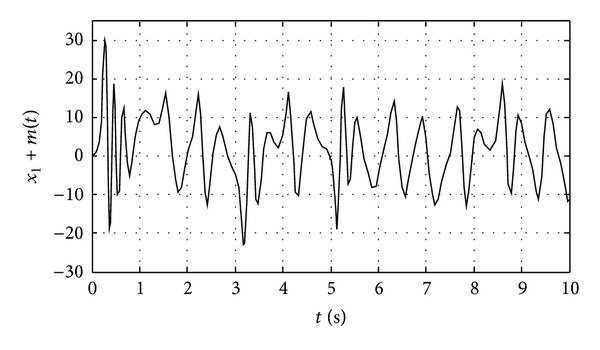
The masked sinusoidal signal.

We apply the proposed chaotic synchronization scheme for secure communication. The plaintext signal is sinusoidal as shown in Figure 3. The state *x*_1_ of the master system is selected as masking signal and the masked encryption information (the signal from transmitter) is shown in Figure 4. It is obvious that the plaintext signal is completely concealed in chaotic signals, and the transmission signal is noise like which results in eavesdroppers' inattention during the information transmission. Because the three states *x*_1_, *x*_2_, and *x*_3_ of the chaos system are all noise like, each of them can be used to mask the sine signal and obtain the equivalent performance.

**Figure 5:**
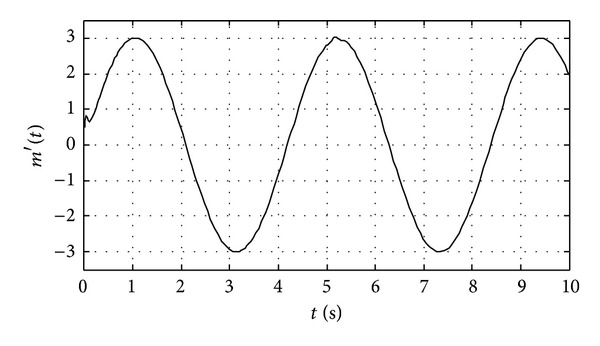
The recovered signal from receiver.

The recovered signal from receiver is shown in Figure 5. We compare the recovered signal with the original signal (in Figure 3) and obviously conclude that the original signal is well decrypted before fault occurring, while it can still be rapidly synchronized and decrypted though the quality of the decrypted signal is affected within a short time after system failure.

**Figure 6:**
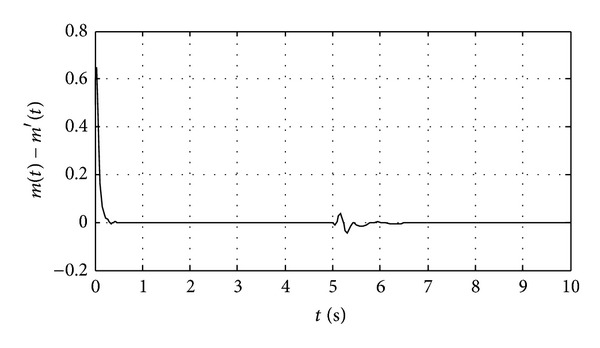
The error curve between plaintext and recovered signal.

The error curve between the plaintext and recovered signal is demonstrated in Figure 6. We can obtain from Figure 6 that the proposed scheme effectively ensures the encryption and decryption of the signal whether system faults occur or not.

## 5. Conclusions

Adopting fault tolerant synchronization method, this paper discusses chaotic fault tolerant synchronization control based on adaptive observer. By utilizing Lyapunov theory of stability, it is proved that the error systems can still get asymptotic stability after system failure. The constructed adaptive observer can not only manage real-time fault diagnosis and parameter identification, but also trace the states of the master system, and so the synchronization between the master system and the slave system is achieved. Then the feasibility and effectiveness of the proposed method are illustrated via numerical simulations of chaotic Chen system.
